# Therapeutic Notes

**Published:** 1909-06-12

**Authors:** 


### THERAPEUTIC NOTES.

### The Active Principle of Oil of Juniper.

The "characters and tests" of oil of juniper as given in the British Pharmacopœia are untrustworthy, and pharmaceutists are endeavouring to define the limits within which genuine oil should fall. At the present time it is to be feared that there is a considerable amount of inferior or adulterated oil on the market—in fact, it is quite possible to make a spurious oil of juniper that will fulfil the Pharmacopœia requirements. The question was thoroughly discussed at the recent meeting of chemists in Manchester, but until it has been decided whether the properties of the oil are due to pinene or to cadinene it will be difficult to fix a standard. In the meantime it would appear advisable to use English oil from a reliable source.

### Dried Extract of Uva Ursi.

Uva ursi has long been used as a diuretic and urinary antiseptic remedy, but chiefly in the form of the infusion. A dried extract of the leaves of uva ursi is now obtainable, and it may be prescribed either by itself or in conjunction with salol, with hexamethylene-tetramine, or with acetyl-salicylic acid. It is conveniently given in cachets, and the drug, either by itself or associated with each of the others mentioned respectively, appears to be the basis of the tablets sold as Uropural, Uropural II., Uropural III., and Uropural IV. The dose of the dried extract of uva ursi is from 4 to 8 grains three times a day, and excellent results are claimed to have been obtained both from it by itself, and from its use along with other antipyuric remedies.

